# Verbal Fluency and Mental-State Recognition in Ecuadorian Adults: Evidence of a Lexical–Cognitive Association in Social Cognition

**DOI:** 10.3390/bs16071215

**Published:** 2026-07-17

**Authors:** Ana Victoria Poenitz, Karen Merizalde, Alexandra Yakeline Meneses Meneses

**Affiliations:** 1 Universidad Tecnológica Israel, Quito 170516, Ecuador; apoenitz@uisrael.edu.ec; 2 Cognitive and Labor Training, Col-Training Group, Pontificia Universidad Católica del Ecuador, Sede Santo Domingo, Santo Domingo 230203, Ecuador; aymenesesm@pucesd.edu.ec

**Keywords:** verbal fluency, social cognition, theory of mind, Reading the Mind in the Eyes Test, neuropsychology, Ecuador, norms

## Abstract

Introduction: Verbal fluency (VF) is a neuropsychological measure sensitive to lexical–semantic organization and prefrontal circuits. Its link with social cognition—specifically with the recognition of mental states from the eyes—remains scarcely explored in Latin American contexts. Objective: To examine the association between performance on the Verbal Fluency Test (phonological and semantic) and mental-state recognition, as measured by the Reading the Mind in the Eyes Test (RMET) in Ecuadorian adults, controlling for sociodemographic variables. Method: A cross-sectional correlational study with 397 healthy adults (Mage = 36.8 years, *SD* = 11.5; range: 18–83; 219 women, 178 men) assessed in Quito, Ecuador, using the multidomain ECUACOG neuropsychological battery. Pearson’s correlations with 95% CIs, Spearman’s correlations, the Kolmogorov–Smirnov test, one-way ANOVA with η^2^, Tukey’s HSD post hoc comparisons, and simple linear regressions were computed. Results: Total VF was significantly associated with RMET score (*r* = 0.227, 95% CI [0.131, 0.319], *p* < 0.001, *N* = 390). Semantic VF showed a higher correlation than phonological VF (*r* = 0.239, 95% CI [0.144, 0.331] vs. *r* = 0.163, 95% CI [0.065, 0.258]; Steiger’s *Z* = 1.86, *p* = 0.063, non-significant trend). Educational level was associated with both VF (semantic VF: η^2^ = 0.093, *p* < 0.001) and RMET (η^2^ = 0.045, *p* < 0.001) scores. The semantic VF–RMET association remained significant after statistically controlling for TMT-B and for educational level (partial *r* = 0.202, *p* < 0.001). Conclusions: Lexical–semantic richness is significantly associated with mental-state recognition in Ecuadorian adults. These findings contribute to the generation of neuropsychological normative data in Ecuador.

## 1. Introduction

Neuropsychological assessment integrates instruments that map different cognitive domains, with verbal fluency (VF) and social cognition being two of the areas of greatest clinical and theoretical relevance. However, their systematic articulation within the context of Ecuadorian neuropsychology has been scarcely investigated. The present study addresses this gap using data from the ECUACOG project, an initiative to standardize neuropsychological instruments in the adult population of Quito, Ecuador.

The relevance of further investigating this relationship extends beyond purely theoretical interest. From an applied perspective, social cognition and verbal fluency are domains frequently compromised—jointly or in dissociation—in clinical conditions such as autism spectrum disorder, schizophrenia-spectrum disorders, acquired brain injury, and early-onset dementia (Lezak et al., 2012). Having normative data that document the expected covariation between both domains in a healthy population is essential for the clinical interpretation of dissociated profiles, in which an isolated deficit in social cognition—in the absence of an equivalent impairment in verbal fluency—could help orient differential diagnosis toward specific conditions within the social-cognitive spectrum. At the academic and social levels, Ecuador lacks its own norms for widely used social-cognition instruments such as the RMET, which forces local clinical practice to rely on foreign norms of uncertain transcultural validity. The ECUACOG project seeks to address this gap, and the present study constitutes a first step toward incorporating the RMET into a normative neuropsychological battery for clinical use in the country.

VF is one of the most widely used neuropsychological tests in clinical and normative assessment (Dorchies et al., 2024). In its phonological modality, it requires generating words beginning with a specified letter within a limited time, which engages fronto-subcortical circuits, lexical retrieval, and executive control (Lezak et al., 2012). The semantic variant, by contrast, requires access to conceptual networks hierarchically organized in semantic memory, with greater reliance on anterior temporal regions and the hippocampus (Mummery et al., 1996). This distinction is not merely theoretical: previous research with Ecuadorian populations—including the normative studies by Rodríguez-Lorenzana et al. (2020) and Poenitz et al. (2022a, 2022b)—has shown that both modalities are sensitive to educational level and age, albeit with differential patterns, suggesting partially independent neurocognitive mechanisms.

For its part, the recognition of mental states from the eyes, assessed using the Reading the Mind in the Eyes Test (RMET; Baron-Cohen et al., 2001), constitutes a measure of affective theory of mind (ToM) that has demonstrated cross-cultural validity and high clinical sensitivity. Recent research has indicated that RMET performance is associated with general linguistic and vocabulary abilities (Dowker & Frye, 2023; Montgomery et al., 2024), suggesting that access to the lexicon of mental states may constitute a necessary requirement for mentalizing. These associations have been documented mainly in healthy English-speaking adult samples, using receptive and expressive vocabulary tests and, to a lesser extent, categorical verbal fluency tasks as linguistic indicators; this leaves unresolved whether the pattern replicates in Spanish-speaking populations and with verbal fluency instruments, such as those used in the ECUACOG battery—an empirical gap the present study seeks to address.

Despite this theoretical convergence, studies that have directly examined the relationship between VF and social cognition in healthy adults are scarce and their results heterogeneous. Studies with non-Latin American samples report small but consistent correlations between both measures (Kynast et al., 2021), whereas in the Spanish-speaking context, data are practically nonexistent. The mechanism proposed to explain this covariation does not refer to the tests themselves, but to the cognitive processes they share: both VF—particularly its semantic modality—and the RMET demand fast and efficient retrieval from a conceptually organized lexicon (taxonomic categories in one case, mental-state terms in the other), as well as executive-control mechanisms for selecting the correct response among competing alternatives (Astington & Baird, 2005; Premack & Woodruff, 1978). Based on this logic, the richness of the mentalistic lexicon available to an individual—which semantic VF would index indirectly through general conceptual organization—would constitute a necessary, although not sufficient, substrate for the explicit attribution of complex mental states.

The objectives of the present study are: (1) to examine the magnitude and significance of the association between VF (phonological, semantic, and total) and RMET score in Ecuadorian adults; (2) to test whether semantic VF predicts RMET performance better than phonological VF using a test for comparing dependent correlations (Meng et al., 1992); (3) to analyze the effect of educational level on both measures using post hoc comparisons; and (4) to discuss the clinical and normative implications of these findings for Ecuadorian neuropsychology.

## 2. Method

### 2.1. Design and Participants

A descriptive-correlational cross-sectional study was conducted. The total ECUACOG project sample consisted of 397 healthy adults (219 women, 178 men) residing in the Metropolitan District of Quito, Ecuador, aged between 18 and 83 years (*M* = 36.8, *SD* = 11.5). Inclusion criteria were: (a) age ≥ 18 years, (b) absence of an active neurological or psychiatric diagnosis, (c) a minimum schooling level of basic education, and (d) native Spanish speakers. Participants with uncorrected visual deficits or a history of severe traumatic brain injury were excluded. The distribution by educational level was: basic education (*n* = 26, 6.5%), high school (*n* = 155, 39.0%), third-level higher education (*n* = 191, 48.1%), and fourth-level higher education (*n* = 18, 4.5%); 7 participants did not report their educational level. Participation was voluntary and informed through written consent.

Verbal Fluency Test data were obtained from *N* = 394 participants; the RMET and the TMT were administered to *N* = 390; the D2 was completed by *N* = 397. The IRI, being the last instrument administered within the assessment session, showed a slightly smaller *N* (*N* = 382): eight participants who did complete the RMET did not go on to complete the IRI, owing to session fatigue or a decision to end the evaluation before the self-report instrument was administered. Missing data resulted from incomplete administration or protocol invalidation per predefined criteria, and showed no systematic pattern (Missing Completely At Random).

### 2.2. Instruments

Verbal Fluency Test (VF): The phonological (generating words beginning with the letter «P» for 60 s) and semantic (naming animals for 60 s) modalities were administered, with preliminary normative data for the Ecuadorian adult population (Poenitz et al., 2022a, 2022b; Rodríguez-Lorenzana et al., 2020). The total number of valid responses per modality was recorded, excluding repetitions and errors. The sum of both modalities constituted the total VF variable.

Reading the Mind in the Eyes Test (RMET): The revised version of the Reading the Mind in the Eyes Test (Baron-Cohen et al., 2001) was administered, comprising 36 photographs of the eye region of adults. For each photograph, the examinee had to select—in a forced-choice, multiple-choice format, with no time limit and no feedback during administration—which of four words (the correct term and three semantically close distractors) best described the mental state of the photographed person. The 36 items span complex mental states of positive, negative, and neutral valence (e.g., reflective, disappointed, or suspicious) in approximately equal proportions, and are not limited to the six basic emotions. The total score (maximum = 36) reflects the ability to infer mental states from minimal visual cues.

Additional ECUACOG Battery Instruments: The following were also administered: (a) the Trail Making Test, parts A and B (TMT; Reitan, 1958); (b) the D2 Test of Attention (Brickenkamp, 2002), using the concentration percentile (CON) as a measure of attentional performance; and (c) the Interpersonal Reactivity Index (IRI; Davis, 1980), across its four subscales. The inclusion of these additional measures served a twofold purpose. On one hand, TMT-B and the D2 concentration percentile allow us to estimate the extent to which the VF–RMET association is independent of processing speed and general attentional control—cognitive functions that covary with verbal fluency and could operate as confounding variables (see the partial-correlation analysis in Section 2.3 and Section 3.2). On the other hand, the IRI, as a trait self-report measure of empathy, allows us to contrast the implicit social cognition assessed by the RMET with subjectively reported empathy—testing the theoretical dissociation between both constructs (Oakley et al., 2016).

### 2.3. Statistical Analysis

Descriptive statistics (mean, standard deviation, minimum, median, and maximum) were calculated for all continuous variables. The normality of distributions was assessed using the Kolmogorov–Smirnov (KS) test; given the violation of this assumption for most variables (phonological VF: KS = 0.092, *p* = 0.003; RMET: KS = 0.109, *p* < 0.001), analyses were complemented with Spearman’s correlations (ρ). Nevertheless, given the sample size (*N* ≥ 382 per instrument), Pearson’s r coefficients are reported as the primary statistic along with 95% confidence intervals calculated via Fisher’s transformation, and Spearman’s ρ values are presented systematically. Associations between the VF and RMET were analyzed using bivariate Pearson’s correlations. A comparison between the correlation coefficients of semantic VF and phonological VF with RMET was performed using Steiger’s test with the Meng et al. (1992) correction for dependent correlations. The effect of educational level on VF and RMET scores was examined using one-way ANOVA, complemented with effect size η^2^ (small ≥ 0.01, medium ≥ 0.06, and large ≥ 0.14; Cohen, 1988) and Tukey’s HSD post hoc comparisons. Simple linear regression was used to estimate the variance in RMET explained independently by semantic VF and phonological VF. To statistically control for the possible confounding effect of sociodemographic and cognitive variables on the VF–RMET association, partial correlations were calculated via ordinary least squares residualization, controlling independently and jointly for: (a) TMT-B, as an indicator of processing speed and general executive function; and (b) educational level, coded as an ordinal variable (1 = basic education, 2 = high school, 3 = third-level higher education, 4 = fourth-level higher education) in the absence of a continuous record of years of formal schooling, complemented with age as an additional covariate. The significance level was set at α = 0.05. Analyses were performed using Python 3.12 (SciPy 1.11).

## 3. Results

### 3.1. Descriptive Statistics

Table 1 presents the descriptive statistics for the study’s main variables. The mean RMET score (*M* = 18.98, *SD* = 5.34) fell within the range reported for healthy Spanish-speaking adults, with a slightly negatively skewed distribution (skewness = −0.84), indicating a tendency toward medium-to-high scores in the sample. Total VF showed a mean of 31.56 words (*SD* = 10.15).

### 3.2. Association Between Verbal Fluency and the Reading the Mind in the Eyes Test

In addition to the main VF–RMET association, we examined the associations between these measures and other instruments in the ECUACOG battery (TMT-B, D2 concentration) in order to contextualize the magnitude of the obtained coefficients (see also Section 3.4). Table 2 summarizes the correlations between pairs of variables of primary interest, including 95% CIs and Spearman’s correlations. Total VF correlated positively and significantly with RMET score (*r* = 0.227, 95% CI [0.131, 0.319], *p* < 0.001, *N* = 390). When the modalities were disaggregated, semantic VF showed a trend toward a higher correlation (*r* = 0.239, 95% CI [0.144, 0.331]) than phonological VF (*r* = 0.163, 95% CI [0.065, 0.258]). The Steiger–Meng test for comparing dependent correlations indicated that this difference does not reach conventional statistical significance (*Z* = 1.86, *p* = 0.063), although it constitutes a trend consistent with the semantic mediation hypothesis. Spearman’s coefficients were practically identical to Pearson’s (ρ_sem = 0.242 and ρ_phon = 0.164), confirming the robustness of the findings against deviations from normality.

Simple linear regression confirmed that semantic VF is a significant predictor of RMET score (standardized β = 0.239, R^2^ = 0.057, *p* < 0.001), whereas phonological VF explained less variance (R^2^ = 0.027, *p* = 0.001). It should be noted that, in simple regression, the standardized β is algebraically equivalent to Pearson’s *r*, so both statistics reflect the same magnitude of association. The unexplained variance (94.3% and 97.3%, respectively) is consistent with the multidetermined nature of both VF and the RMET, and underscores that the reported associations are small but statistically robust.

To estimate the extent to which the VF–RMET association is independent of processing speed, general executive function, and educational level—the latter associated with both VF and RMET (see Section 3.3)—partial correlations were calculated controlling statistically for TMT-B, for educational level (coded as an ordinal variable from 1 to 4), and for both variables jointly (Table 3). The semantic VF–RMET association remained significant after controlling for TMT-B (partial *r* = 0.223, *p* < 0.001), for educational level (partial *r* = 0.228, *p* < 0.001), and when controlling simultaneously for both variables (partial *r* = 0.202, *p* < 0.001), with modest reductions relative to the bivariate coefficient (*r* = 0.239). The same pattern was observed for total VF–RMET (partial *r* ranging from 0.186 to 0.209, all *p* < 0.001) and for phonological VF–RMET (partial *r* = 0.144, *p* = 0.005, controlling for TMT-B). Additionally, controlling for age together with educational level did not substantially change the coefficients (semantic VF–RMET, partial *r* = 0.227, *p* < 0.001). Taken together, these results indicate that the association between verbal fluency—particularly its semantic component—and mental-state recognition is not attributable solely to differences in processing speed, general executive function, or schooling, although these variables account for a non-trivial portion of the raw covariation observed.

### 3.3. Effect of Educational Level on Verbal Fluency and the Reading the Mind in the Eyes Test

Educational level showed a significant effect on phonological VF (*F* [3, 386] = 16.77, *p* < 0.001, η^2^ = 0.115), semantic VF (*F* [3, 386] = 13.12, *p* < 0.001, η^2^ = 0.093), and RMET score (*F* [3, 385] = 6.07, *p* < 0.001, η^2^ = 0.045). Tukey’s HSD post hoc comparisons revealed that, for phonological VF, the high-school group scored significantly lower than the third-level higher education group (*p* < 0.001) and the fourth-level higher education group (*p* < 0.001). For semantic VF, the fourth-level group differed significantly from all other groups (*p* < 0.01), and the third-level group scored higher than the high-school group (*p* = 0.003) and the basic-education group (*p* = 0.009). For RMET, the basic-education group scored significantly lower than the three remaining groups (basic vs. high school: *p* = 0.011; basic vs. third level: *p* < 0.001; basic vs. fourth level: *p* = 0.006), whereas the three groups with intermediate and higher schooling did not differ from one another. Table 4 details the means, standard deviations, and results by group.

### 3.4. Concurrent Correlations and IRI-RMET Dissociation

Total VF was significantly associated with TMT-B (*r* = 0.266, 95% CI [0.171, 0.356], *p* < 0.001) and with D2 concentration (*r* = 0.195, 95% CI [0.097, 0.288], *p* < 0.001). RMET score also showed a significant correlation with D2 concentration (*r* = 0.185, 95% CI [0.088, 0.280], *p* < 0.001). In contrast, none of the IRI subscales correlated significantly with RMET score (Table 5)—a dissociation consistent with the theoretical distinction between implicit social cognition (ToM) and self-reported empathy.

## 4. Discussion

The present study examines, for the first time with a large sample of Ecuadorian adults (*N* = 397), the relationship between verbal fluency (VF) and mental-state recognition as measured by the RMET (Baron-Cohen et al., 2001). The results confirm the main hypothesis: total VF is significantly and positively associated with RMET performance (*r* = 0.227, 95% CI [0.131, 0.319], *p* < 0.001). Semantic VF showed a trend toward a higher correlation than phonological VF (*r* = 0.239 vs. *r* = 0.163), although the formal comparison using the Steiger–Meng test did not reach conventional statistical significance (*Z* = 1.86, *p* = 0.063); this precludes categorically affirming the superiority of the semantic modality, but it does indicate a trend consistent with the organized conceptual access hypothesis. This association remained significant, albeit somewhat attenuated, after statistically controlling for processing speed/general executive function (TMT-B) and for educational level (Table 3), suggesting that the VF–RMET link is not reducible to a third-variable cognitive or sociodemographic effect.

### 4.1. Contextualization Within the Existing Literature

The association between linguistic competence and mentalizing ability has been documented in the neuropsychological literature. Dowker and Frye (2023) demonstrated that ToM abilities are robustly associated with indicators of expressive and receptive language. Montgomery et al. (2024) reported that language comprehension and executive function measures independently contributed to predicting performance on affective and cognitive perspective-taking tasks. Pathare et al. (2025) propose that language acts as a substrate of the reflective–explicit system of mental-state attribution.

The small R^2^ coefficients obtained (semantic VF: R^2^ = 0.057; phonological VF: R^2^ = 0.027) are consistent with the international literature (Kynast et al., 2021), and should be interpreted within the context of the simple correlational design: the study’s aim was to establish the existence and direction of the association, not its full magnitude. The unexplained variance reflects the multidetermined nature of both functions, including factors such as working memory and fluid intelligence, which were not directly measured in the present battery. Statistical control for TMT-B (Table 3) suggests that general processing speed explains only a modest portion of the VF–RMET covariation, pointing the remaining variance more specifically toward lexical–semantic processes.

These findings align with the proposal by Pathare et al. (2025) that language operates as a substrate of the reflective–explicit system of mental-state attribution, rather than as a merely incidental correlate of social cognition. The persistence of the semantic VF–RMET association after controlling for TMT-B and educational level reinforces this interpretation: if the covariation were entirely explained by general differences in cognitive efficiency or schooling, it would have nearly disappeared once these controls were introduced, which was not the case. At the same time, the magnitude of the coefficients—small according to Cohen’s (1988) conventions—indicates that lexical–conceptual access constitutes, at most, a partial facilitating condition for mentalizing, rather than its principal determinant; other components (e.g., context-based social inference and emotional regulation) likely contribute independently to RMET performance.

### 4.2. The Effect of Educational Level

The educational gradient was especially pronounced for semantic VF (η^2^ = 0.093), with a +77.4% increase between basic education (*M* = 12.35) and the fourth level (*M* = 21.89). Tukey’s HSD post hoc analyses revealed that this gradient is mainly driven by the fourth-level group, which differs significantly from all other groups. For RMET, the significant difference is concentrated between basic education and the rest, with no differences among the three groups with intermediate and higher schooling levels, suggesting that a minimal schooling threshold (rather than a continuous gradient) predicts ToM performance.

### 4.3. IRI-RMET Dissociation and Limitations

The absence of significant correlations between the IRI subscales and RMET (all *p* > 0.05; Table 5) is consistent with the proposal by Oakley et al. (2016) that the RMET measures cognitive ToM rather than emotional empathy.

The present study has several limitations. First, the cross-sectional design does not allow establishing the causal direction of the reported associations. Second, the sample is limited to the Metropolitan District of Quito, restricting representativeness for plurinational Ecuador. Third, the RMET was administered without formal adaptation to Ecuadorian Spanish. This absence of formal adaptation is not merely a procedural detail: English terms describing complex mental states (e.g., *reflective*, *contemplative,* or *fantasizing*) do not necessarily have everyday lexical equivalents of identical connotation in Ecuadorian Spanish, so the four response options for each item may not be equally discriminable for Spanish speakers as for native English speakers. This cross-cultural non-equivalence of mental-state terms could introduce a measurement bias of uncertain direction—overestimating the difficulty of some items and underestimating that of others—and constitutes, in itself, an additional argument for undertaking a formal linguistic and conceptual validation of the RMET for Ecuadorian Spanish before its extended normative use. Finally, the absence of direct measures of working memory and fluid intelligence precludes fully ruling out their contribution to the semantic VF–RMET association; although statistical control via TMT-B (Table 3) indicates that processing speed and general executive function explain only part of the covariation, both remaining constructs were not directly measured in the present battery. Future research using multiple regression models or structural equation modeling could address these limitations.

## 5. Conclusions

The present study demonstrates that verbal fluency—particularly its semantic component—is significantly associated with mental-state recognition as assessed by the Reading the Mind in the Eyes Test in urban Ecuadorian adults, an association that remained significant after statistically controlling for processing speed/general executive function and for educational level. Semantic VF showed a trend toward a higher association than phonological VF, although the difference between the two coefficients did not reach conventional statistical significance (Z = 1.86, *p* = 0.063), indicating a trend consistent with the organized conceptual access hypothesis. Tukey’s HSD post hoc tests revealed that the educational effect on the RMET score is concentrated mainly in the contrast between basic education and higher educational levels. The inclusion of the RMET in the ECUACOG battery and the availability of normative data stratified by schooling will strengthen diagnostic precision in Ecuadorian clinical neuropsychology.

## Figures and Tables

**Table 1:** Descriptive statistics for the main variables.

Variable/Instrument	*N*	*M*	*SD*	Min	*Mdn*	Max
Phonological Verbal Fluency	394	16.04	4.76	4	17	30
Semantic Verbal Fluency	394	15.52	6.38	1	16	30
Total Verbal Fluency (VF-T)	394	31.56	10.15	6	33	58
Reading the Mind in the Eyes Test—score	390	18.98	5.34	2	20	28
TMT-A (seconds)	390	44.35	22.91	16	37	139
TMT-B (seconds)	390	85.31	43.98	21	75	363
D2—Concentration (CON, percentile)	397	57.91	30.77	1	60	170

*Note. M* = arithmetic mean; *SD* = standard deviation; *Mdn* = median. VF = verbal fluency; RMET = Reading the Mind in the Eyes Test; TMT = Trail Making Test; D2-CON = D2 concentration (percentile). VF: *N* = 394; RMET, TMT-A, TMT-B: *N* = 390; D2-CON: *N* = 397.

**Table 2:** Pearson’s correlations with 95% CIs and Spearman’s correlations among neuropsychological variables (*N* = 390).

Variable Pair	*r*	95% CI	*N*	*p*	ρ	Interpretation
*Semantic VF → RMET*	0.239	[0.144, 0.331]	390	<0.001 ***	0.242	Moderate positive
*Phonological VF → RMET*	0.163	[0.065, 0.258]	390	0.001 **	0.164	Small positive
*Total VF → RMET*	0.227	[0.131, 0.319]	390	<0.001 ***	0.228	Moderate positive
*Total VF → TMT-B*	0.266	[0.171, 0.356]	390	<0.001 ***	0.233	Moderate positive
*Total VF → D2-CON*	0.195	[0.097, 0.288]	390	<0.001 ***	0.186	Small–moderate
*TMT-A → TMT-B*	0.505	[0.427, 0.575]	390	<0.001 ***	0.511	Moderate–high
*RMET → D2-CON*	0.185	[0.088, 0.280]	390	<0.001 ***	0.174	Small–moderate

*Note*: 95% CIs calculated using Fisher’s transformation. ** *p* < 0.01; *** *p* < 0.001. VF = verbal fluency; RMET = Reading the Mind in the Eyes Test; TMT-B = Trail Making Test, part B; D2-CON = D2 concentration. The interpretation column strictly classifies the magnitude of *r* following Cohen’s (1988) conventions: small ≈ 0.10, moderate ≈ 0.30, large ≈ 0.50.

**Table 3:** Partial correlations between verbal fluency and RMET, controlling for TMT-B and educational level (*N* = 390).

Variable Pair	*r*	Control Variable(s)	Partial *r*	*N*	*p* (Partial)
*Semantic VF → RMET*	0.239	TMT-B	0.223	390	<0.001 ***
*Total VF → RMET*	0.227	TMT-B	0.209	390	<0.001 ***
*Phonological VF → RMET*	0.163	TMT-B	0.144	390	0.005 **
*Semantic VF → RMET*	0.239	Educational level	0.228	390	<0.001 ***
*Total VF → RMET*	0.227	Educational level	0.208	390	<0.001 ***
*Semantic VF → RMET*	0.239	Educational level + age	0.227	390	<0.001 ***
*Semantic VF → RMET*	0.239	TMT-B + educational level	0.202	390	<0.001 ***
*Total VF → RMET*	0.227	TMT-B + educational level	0.186	390	<0.001 ***

*Note*: Partial correlations were calculated via ordinary least squares residualization. ** *p* < 0.01; *** *p* < 0.001. VF = verbal fluency; RMET = Reading the Mind in the Eyes Test; TMT-B = Trail Making Test, part B. Educational level was coded as an ordinal variable (1 = basic, 2 = high school, 3 = third level, 4 = fourth level).

**Table 4:** Means (standard deviations) by educational level, one-way ANOVA results, and Tukey’s HSD post hoc comparisons.

Dependent Variable	Basic (*n* = 26)	High School (*n* = 155)	Third Level (*n* = 191)	Fourth Level (*n* = 18)	*F*(3, k)	*p*/η^2^	Post Hoc
Phonological VF	15.81 (6.20)	14.16 (4.59)	17.16 (3.99)	19.61 (5.30)	16.77	<0.001/0.115	BAS > HS ***; BAS > T3 ***; T4 > HS *
Semantic VF	12.35 (8.23)	14.06 (6.00)	16.36 (5.80)	21.89 (5.07)	13.12	<0.001/0.093	T4 > all **; T3 > HS **; T3 > BAS **
Reading the Mind in the Eyes Test	15.92 (6.59)	18.84 (5.81)	19.60 (4.33)	20.65 (4.64)	6.07	<0.001/0.045	BAS < HS *; BAS < T3 ***; BAS < T4 **

*Note*: Values in parentheses correspond to the standard deviation. *F* = *F* statistic from the one-way ANOVA, with degrees of freedom in parentheses; η^2^ = eta squared. *** *p* < 0.001; ** *p* < 0.01; * *p* < 0.05. BAS = basic; HS = high school; T3 = third level; T4 = fourth level. The symbol > indicates that the group on the left obtained a significantly higher mean.

**Table 5:** Correlations between IRI subscales and RMET score (*N* = 382).

IRI Subscale → RMET	*r*	95% CI	*N*	*p*	ρ	Interpretation	Sig.
Perspective Taking	0.027	[−0.073, 0.127]	382	0.597	—	No association	ns
Fantasy	0.047	[−0.054, 0.146]	382	0.364	—	No association	ns
Empathic Concern	0.022	[−0.079, 0.122]	382	0.672	—	No association	ns
Personal Distress	−0.016	[−0.116, 0.084]	382	0.752	—	No association	ns

*Note*: 95% CIs calculated using Fisher’s transformation. ns = not significant. IRI = Interpersonal Reactivity Index; RMET = Reading the Mind in the Eyes Test.

## Data Availability

The anonymized data supporting the findings of this study are available upon reasonable request to the corresponding author (apoenitz@uisrael.edu.ec). The authors commit to depositing the anonymized dataset in an open-access repository (OSF or Zenodo) during the peer-review stage.
